# The tripartite interactions between the mosquito, its microbiota and *Plasmodium*

**DOI:** 10.1186/s13071-018-2784-x

**Published:** 2018-03-20

**Authors:** Ottavia Romoli, Mathilde Gendrin

**Affiliations:** 1 Microbiota of Insect Vectors Group, Institut Pasteur de la Guyane, Cayenne, French Guiana France; 2 Parasites and Insect Vectors Department, Institut Pasteur, Paris, France

**Keywords:** Microbiota, *Anopheles*, *Plasmodium*, Experimental models, Vectorial transmission, Colonisation resistance

## Abstract

The microbiota of *Anopheles* mosquitoes interferes with mosquito infection by *Plasmodium* and influences mosquito fitness, therefore affecting vectorial capacity. This natural barrier to malaria transmission has been regarded with growing interest in the last 20 years, as it may be a source of new transmission-blocking strategies. The last decade has seen tremendous progress in the functional characterisation of the tripartite interactions between the mosquito, its microbiota and *Plasmodium* parasites. In this review, we provide insights into the effects of the mosquito microbiota on *Plasmodium* infection and on mosquito physiology, and on how these aspects together influence vectorial capacity. We also discuss three current challenges in the field, namely the need for a more relevant microbiota composition in experimental mosquitoes involved in vector biology studies, for a better characterisation of the non-bacterial microbiota, and for further functional studies of the microbiota present outside the gut.

## Background

*Plasmodium* malaria parasites are transmitted by *Anopheles* mosquitoes. Female mosquitoes become infected after taking a blood meal on humans carrying *Plasmodium* pre-sexual stages. Upon entering the gut lumen, parasites undergo sexual reproduction and differentiate into motile forms, ookinetes, within 24 hours. After crossing the gut epithelium, the ookinete develops into an oocyst which undergoes mitoses in the following week and releases sporozoites in the hemolymph. About 10–14 days after the infected blood meal, sporozoites reach the salivary glands. The mosquito then becomes infectious and will inject parasites to humans with its saliva during subsequent bites for the rest of its life.

Ingestion of an infectious blood meal will only result in malaria transmission if the parasite makes it through bottlenecks in the gut and salivary glands and if the mosquito bites humans after the extrinsic incubation period, the time needed for parasites to become infectious. The gut, salivary glands and reproductive organs are colonised by a dynamic microbial community composed by bacteria, viruses and fungi, of which the bacterial part is the best characterised [1–3]. This microbiota impacts disease transmission by interfering with *Plasmodium* colonisation in the gut and by affecting different aspects of mosquito physiology, notably its lifespan.

As a consequence, microorganisms that colonise the mosquito are regarded as potential tools to reduce malaria transmission. They may be used to shorten the mosquito lifespan or to decrease *Plasmodium* infection rates, either *via* natural competition mechanisms [4, 5] or *via* the production of genetically introduced anti-*Plasmodium* molecules, so called paratransgenesis [6–8]. One of the main advantages of a strategy based on microbial colonisation is the potential for targeting several species of mosquitoes and *Plasmodium* at the same time.

During development, the mosquito acquires its microbiota from its mother’s genitalia and from its larval and pupal breeding site. Some of these microorganisms are trans-stadially transmitted to the adult [9, 10], while others are acquired by adults when feeding on different substrates or during mating [11]. The microbiota population is particularly dynamic in the mosquito gut, where it drastically expands after a blood meal. More specifically, this proliferation is observed in the middle region of the gut, the midgut, where the blood is stored during digestion over a 2-day period and where the early stages of *Plasmodium* development take place. Here, we review the current knowledge on tripartite interactions between the mosquito, its microbiota and *Plasmodium* and we discuss current challenges of the field.

## Effects of the microbiota on vectorial capacity

Since the midgut represents the first and main bottleneck of parasite development, the microbial community present in its lumen has a strong role in the first stages of *Plasmodium* infection. Several functional studies have investigated the specific role of the midgut microbiota on *Plasmodium* infection. These studies, performed on five *Anopheles* species and four *Plasmodium* species, point to an overall inhibitory effect of the microbiota on the parasite, independent of the species of *Anopheles* and *Plasmodium* (Table 1). The effect is, however, highly bacterial strain specific [4, 12–14]. Most bacteria showing an anti-parasitic effect are Gram-negative [12, 14].

**Table 1:** Effects of the microbiota or of specific bacterial species/strains on *Plasmodium* infection in different *Anopheles* mosquitoes in chronological order

Mosquito	Parasite	Inhibition	No inhibition	Reference
*An. stephensi* (colony)	*P. falciparum*	*Escherichia coli* H243^a^; *Pseudomonas aeruginosa*^a^; *Ewingella americana*^a^	*Staphylococcus aureus*^b^; *S. epidermidis*^b^; *E. coli* HB101^a^	[12]
*An. stephensi* (colony)	*P. falciparum*	*E. coli* HS5^a^; *P. aeruginosa*^a^; *Serratia marcescens*^a^; *Xanthomonas malthophila*^a^; *Cedecea lapagei*^a^		[67]
*An. albimanus* (colony)	*P. vivax*	*S. marcescens*^a^; *Enterobacter cloacae*^a^; *Enterobacter amnigenus* 2^a^		[4]
*An. gambiae*^c^ (colony)	*P. falciparum*	Microbiota; Live and heat-inactivated mixture of *S. aureus*^b^ *+ E. coli*^a^		[5]
*An. gambiae*^d^, *An. coluzzii* (colonies)	*P. berghei*	*S. aureus*^b^; *E. coli*^a^; *E. cloacae*^a^		[15]
*An. gambiae*^c^, *An. stephensi* (colonies)	*P. falciparum*	*Enterobacter* sp*.* Zambia^a^	*Bacillus pumilus* ^b^	[13]
*An. gambiae*^d^ (colony)	*P. yoelii*		Microbiota	[68]
*An. gambiae*^d^ (colony)	*P. falciparum*	Microbiota		
*An. coluzzii* (colony)	*P. falciparum*	*E. coli*^a^; *S. marcescens*^a^; *Pseudomonas sutzeri*^a^; *Comamonas* spp.^a^; *Enterobacter* spp.^a^; *B. pumilus*^b^	*Acinetobacter septicus* ^a^	[69]
*An. stephensi* (colony)	*P. berghei*	*S. marcescens* HB3^a^	*S. marcescens* HB18^a^	[14]
*An. dirus* (colony)	*P. yoelii*	Microbiota		[19]
*An. gambiae*^c^ (colony)	*P. falciparum*	*Chromobacterium* sp*.* Csp_P^a^		[22]
*An. gambiae*^c^ (colony)	*P. falciparum*	*P. putida*^a^; *Pantoea* sp.^a^; *S. marcescens*^a^		[16]
*An. gambiae*^e^ (from field larvae)	*P. falciparum*	*Serratia*^a^; *Methylobacterium*^a^		[3]

^a^Gram-negative bacteria

^b^Gram-positive bacteria

^c^Keele strain, hybrid of *An. gambiae* and *An. coluzzii*

^d^G3 strain, hybrid of *An. gambiae* and *An. coluzzii*

^e^Field-collected mosquitoes, not specified if *An. gambiae* or *An. coluzzii*

### Antiparasitic effects of the microbiota on *Plasmodium*

**Fig. 1:**
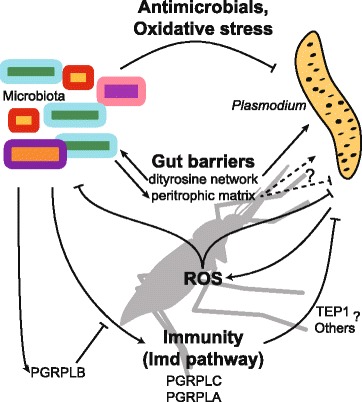
Interactions between the microbiota and *Plasmodium* in the mosquito midgut. The microbiota affects *Plasmodium* infection by several mechanisms: (i) Direct impact on parasites *via* inhibition of its oxidative defence system [13, 20] or by production of uncharacterised antimicrobials [16, 22]. (ii) Stimulation of the NF-κB dependent Immune-deficiency (Imd) pathway, which is regulated by Peptidoglycan Recognition Proteins (PGRPs) and restrains parasite infection [15, 30]. The mechanisms of action of the Imd pathway on parasites are still unclear, they probably include TEP1-dependent and independent components [5, 18, 19]. (iii) Blood meal inducible physical barriers affect gut microbes: a dityrosine network reduces the diffusion of elicitors, thus protecting the microbiota and *Plasmodium* from immune activation [32] and the microbiota-dependent induction of the peritrophic matrix [24] may have positive and/or negative impacts on parasite infection. Reciprocally, *Plasmodium* infection inhibits antioxidant enzymes in the mosquito gut, which has been suggested to help parasite infection *via* a reduction of the mosquito microbiota [21]

The microbiota interferes with *Plasmodium* colonisation of the mosquito gut through at least two mechanisms: (i) stimulation of the mosquito immune response; and (ii) production of metabolites directly impairing parasite survival (Fig. 1).

The fast multiplication of bacteria in the gut following a blood meal stimulates an immune response which is widely antimicrobial. At the level of the midgut epithelium, this immune response is largely due to the Immune-deficiency (Imd) pathway, which is induced upon detection of the bacterial cell wall peptidoglycan by Peptidoglycan Recognition Protein (PGRP) LC and positively regulated by PGRPLA [15–17]. How the Imd pathway affects the parasite has not yet been characterised, but this pathway has been shown to induce *TEP1* expression, and TEP1 has been reported to participate in the microbiota-dependent control of *Plasmodium* infection [18, 19]. Ookinete numbers are, however, already reduced in the gut epithelium in a microbiota-dependent manner, suggesting that microbiota-induced immunity also affects parasites before the action of the complement system [5].

Some microorganisms isolated from the mosquito gut also produce metabolites that directly affect *Plasmodium* and reduce its ability to infect the mosquito. *Enterobacter* Esp_Z is a Gram-negative bacterium isolated from the gut of *An. arabiensis* mosquitoes that was found to reduce *P. falciparum* ookinete, oocyst and sporozoite loads in *An. gambiae via* production of reactive oxygen species and/or inhibition of the oxidative defence system of the parasite [13, 20]. Interestingly, *Plasmodium* infection has been reported to reciprocally inhibit antioxidant enzymes in the mosquito gut. This may promote parasite infection *via* a reduction of the mosquito microbiota [21]. Some *Serratia marcescens* strains and *Chromobacterium* Csp_P initially isolated from the mosquito midgut reduce *P. falciparum* infection in *An. gambiae* and show anti-parasitic activity *in vitro* by producing one or several uncharacterised metabolites [16, 22]. Moreover, the yeast *Wickerhamomyces anomalus* was isolated from the midgut of *An. stephensi* and found to produce a killing toxin with β-1,3-glucanase activity, which inhibits *P. berghei* ookinetes *in vitro* [23].

Besides these well-characterised effects of the microbiota on *Plasmodium*, some effects are indirectly suggested in the literature. For instance, the microbiota is also involved in the synthesis of the peritrophic matrix, a layer composed of chitin and proteins which surrounds the midgut epithelium after blood-feeding and protects the mosquito from the dissemination of midgut bacteria into the body cavity [24]. *Plasmodium* ookinetes secrete a chitinase required to cross the peritrophic matrix [25, 26], indicating that this barrier, if not impenetrable for malaria parasites, exerts at least some selection pressure on them. From this point-of-view, it may be a colonisation resistance mechanism induced by the microbiota against *Plasmodium*.

The microbiota may also nutritionally affect *Plasmodium* in the mosquito gut. Whole genome sequencing of bacterial strains of the mosquito microbiota identified genes involved in the digestion of macromolecules [27]. However, it is yet unknown whether this potential digestive role of the microbiota results in a benefit for the mosquito and for *Plasmodium* or in nutrient competition. In *Drosophila*, resistance to virus infection induced by endosymbionts is at least partly due to competition for cholesterol [28]. Nutrient availability is manipulated by *Plasmodium* in the mosquito gut through overexpression of digestive enzymes, suggesting that the high nutritional requirement of the parasite at its early developmental stages is a limiting factor for host colonisation [29].

### Positive effects of the microbiota on *Plasmodium*

Several tolerance mechanisms avoid chronic activation of the immune system by gut microorganisms and participate in maintaining gut homeostasis, but they may also impair the mosquito immune defence against the parasite. Notably, PGRPLB is a negative regulator of the Imd pathway preventing systemic antimicrobial responses to the microbiota, which participates in a higher tolerance to *Plasmodium* infection [30]. Its expression is induced by the microbiota [24, 31]. Immunomodulatory Peroxidase (IMPer) and Dual oxidase (Duox) enzymes, induced by the blood meal in a microbiota-independent manner [24, 31], are involved in the formation of a dityrosine network between the gut epithelium and the peritrophic matrix [32]. This reduction in permeability protects both the microbiota and *Plasmodium* by preventing the activation of gut immunity. Whether the microbiota-induced peritrophic matrix [24] further reduces the diffusion of immune elicitors and is also involved in tolerance mechanisms has not yet been characterised. Along these lines, antibiotic treatment leads to an increase in the diffusion of 4kD dextran molecules from the gut to the body cavity even in IMPer-silenced mosquitoes [32].

Finally, the microbiota is also reported to contribute to the nutrition of the mosquito and *Plasmodium*. After feeding *An. stephensi* with [^14^C]-glycine radiolabelled *Pseudomonas* isolated from the *Anopheles* microbiota, radioactivity is detectable not only in the whole mosquito body, but also in *P. berghei* oocysts and in disseminated sporozoites [33]. This may reflect a positive effect of the microbiota on proliferation within the oocyst.

So far, antibiotic treatments and bacterial feeding treatment have reproducibly shown that the microbiota reduces infection by *Plasmodium* (Table 1), suggesting that even though some tolerance pathways and nutritional benefits exist, they only lessen the overall negative impact of the microbiota on *Plasmodium*.

### Impact of the microbiota on mosquito fitness

**Fig. 2:**
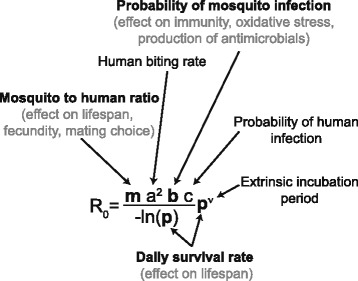
The microbiota impacts several parameters of the Ross-MacDonald model of vectorial transmission. R_0_ represents the basic reproductive number, the number of individuals that are expected to get infected *via* mosquito transmission when a single infected individual is present in a susceptible population. The variables indicated in bold are known to be microbiota-dependent. In grey, the specific effects of the microbiota on mosquito physiology and immunity are specified [13, 15–17, 20, 22, 23, 30, 35]. Potential roles of the mosquito microbiota on biting rate, anthropophily, incubation period and probability of human infection have not yet been investigated

A variety of factors influence parasite transmission by the mosquito. An estimation of their relative contribution to vectorial capacity is provided by the Ross-Macdonald model, which quantifies the basic reproductive number R_0_, i.e. the expected number of secondary infections from a single infected individual in a susceptible population (Fig. 2; [34]). It depends on the population sizes of mosquitoes and humans, the biting rate on humans, the success of parasite infection, the incubation period of the parasite in the mosquito and the mosquito lifespan.

As shown in Fig. 2, the microbiota impacts R_0_ *via* different aspects of mosquito fitness such as insect development, lifespan, fecundity and mating behaviour [10, 17, 35]. More specifically, the microbiota is fundamental for mosquito development: *Ae. aegypti* and *An. gambiae* larvae depleted of their gut microbes are unable to develop into adults [10]. A reduction in the proliferation of gut bacteria *via* a mild antibiotic treatment of the blood meal in *An. coluzzii* increases the mosquito fecundity and survival, meaning that the natural microbiota proliferation potentially decreases mosquito population size and time available for parasite transmission [17]. Some bacteria isolated from the midgut of *Anopheles* were shown to decrease mosquito lifespan when individually administered through sugar or blood meal while others have no effect [16, 20, 22]. Although the abundance and the effect of these single bacteria likely differ in physiological conditions, these studies suggest a composition-dependent negative impact of the gut microbiota on lifespan. A recent study reported a modification of the *An. stephensi* microbiota following manipulation of mosquito immunity and showed that this in turn influences reproduction behaviour, by increasing the chance of mating between mosquitoes harbouring a different microbiota [35]. All of these aspects may alter the probability of transmitting the *Plasmodium* parasite and thus have a strong impact on vector control.

## Current challenges in the study of the mosquito microbiota

### Working on mosquitoes with a representative microbiota composition

The *Anopheles* midgut hosts a simple, variable and dynamic microbial community whose composition mostly depends on environmental factors and individual history. More particularly, seasonality, diet, larval breeding site and blood-feeding history, but also host genetic identity, have a strong influence in shaping the midgut bacterial content [1, 3, 36–41]. The high variability of those factors in the field probably explains the high diversity of the mosquito microbiota composition between individuals, almost independently of the mosquito species [10, 36, 42–46]. Different studies suggest that the mosquito microbiota composition is not random, but whether a core bacterial community exists is still not clear. On one side, some bacterial genera are frequently found in *Anopheles* midguts. They are Gram-negative aerobic or facultative aerobic bacteria, mostly belonging to the families *Enterobacteriaceae* (*Serratia*, *Ewingella*, *Enterobacter* and *Klebsiella*), *Acetobacteraceae* (*Acetobacter* and *Asaia*) and *Flavobacteriaceae* (*Elizabethkingia* and *Chryseobacterium*) (reviewed in [47]). On the other side, the bacterial diversity is relatively low, with four operational taxonomic units (OTUs) representing more than 90% of the midgut community and a poor composition overlap between individual mosquitoes [45]. This suggests that, rather than a core microbiota, several typical microbiota compositions, or enterotypes, may be defined in *Anopheles* mosquitoes. Such types may derive from individual history, environment and host genetics.

**Fig. 3:**
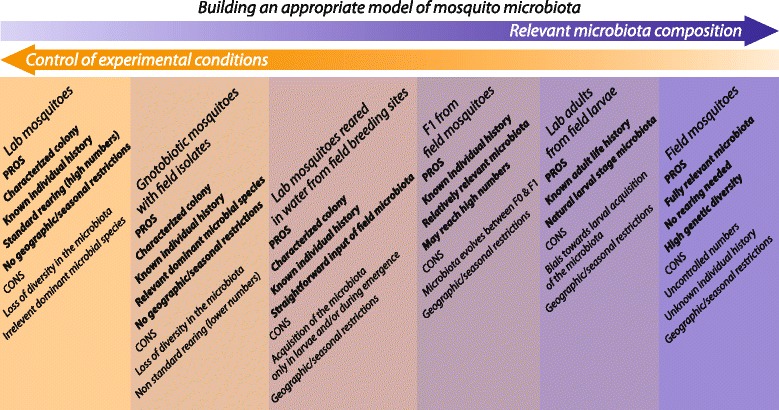
Alternative models for the study of the mosquito and its microbiota. Besides laboratory conventionally-reared mosquitoes and field-collected mosquitoes, several other microbiota set ups are or may be used to study the mosquito biology. Field bacterial isolates may be used to obtain gnotobiotic mosquitoes, i.e. mosquitoes colonised by known strains of bacteria. Laboratory larvae may be reared in water collected from natural breeding sites, or a first generation of mosquitoes (F1) may be obtained from field collected individuals. Finally, adult mosquitoes may be raised in the laboratory from field collected larvae. None of these models is perfect but each may be more appropriate depending on the aims and requirements of each study. Combining several models may also strengthen experimental work and participate in increasing the reproducibility of results between laboratories

Remarkably, *Anopheles* reared in laboratory conditions showed a reduced bacterial diversity in their midgut with respect to field-collected ones, although the majority of bacteria found in laboratory mosquitoes were present in wild mosquitoes [1, 36, 43]. Functional studies on the interactions between the microbiota and *Plasmodium* have mostly been conducted on mosquitoes reared under controlled laboratory conditions (Table 1). The microbiota composition of laboratory-reared mosquitoes varies between insectaries due to local variations and differences in husbandry [48]. This variability could be one of the explanations for some discrepancies of results between laboratories in vector biology. Meanwhile, microbiota studies on field-caught mosquitoes identified correlations, with *Plasmodium* infection for instance, but are elusive on the causes and consequences of these correlations. Notably, Straif et al. [49] found an association between Gram-positive bacteria and *P. falciparum* infection in field-collected *An. funestus*, while Boissière et al. [36] and Tchioffo et al. [3] found a positive correlation between parasite infection and *Enterobacteriaceae* or *S. marcescens* (Gram-negatives) abundance in *An. gambiae* after experimental infection of adults derived from field-collected larvae. These data point out the need for more appropriate microbiota models to perform functional studies on the tripartite relationship between the mosquito, the microbiota and *Plasmodium*. Several experimental setups may be favoured and are shown in Fig. 3 with their pros and cons. Choosing the most appropriate model to answer a scientific question, or combining the use of several models, may strengthen vector biology studies.

### Further identification of the role of the non-bacterial microbiota

Despite the high number of studies on the midgut microbiota, our knowledge of its non-bacterial components is still poor. Metagenomic data on total mosquito RNA detected several viruses associated with wild-caught *Anopheles* [50, 51]. Similar data from other mosquitoes also catalogued some viral, bacterial and eukaryotic components of the microbiota [2], but cost and computational requirements limit the number of samples analysed.

The analysis of the eukaryotic microbiota by *18S* rRNA sequencing is in part complicated by the amplification of DNA from the mosquito or from the blood source [52]. Several fungi belonging to the genera *Candida*, *Pichia* and *Penicillium* were isolated from *Anopheles* larvae and adults through standard microbiological techniques [53–55]. Two functional studies reported that specific fungi isolated from mosquitoes influence *Plasmodium* infectiveness either negatively [56] or positively [57]. However, we still lack an exhaustive investigation of the viral and eukaryotic components of the mosquito microbiota and of their interactions with *Plasmodium.*

Along these lines, the parasite itself may be considered as part of the microbial community, or pathobiome [58], harboured by the mosquito. In other insect-pathogen models, it has been shown that the invading microorganism can induce significant changes in the host microbiota composition to increase its infection success. For example, when the human pathogen *Anaplasma phagocytophilum* infects the tick *Ixodes scapularis*, it induces significant variations in the composition of the host gut microbial community. This correlates with pathogen-dependent induction of the *Ixodes* Antifreeze Glycoprotein IAFGP, which reduces biofilm formation and impairs the integrity of the peritrophic matrix, facilitating penetration of the pathogen into the tick body cavity [59]. Since similar interactions have not been described for the *Anopheles-*microbiota-*Plasmodium* system, further studies are required to clarify the role of the parasite in influencing the microbiota and host immunity.

### Widen the knowledge on the microbiota in other tissues

The microbial communities colonising the mosquito salivary glands and reproductive organs are as relevant as those in the midgut for several aspects concerning *Plasmodium* transmission and mosquito ecology. Microorganisms present in the salivary glands are prone to interact with the parasite when the mosquito is becoming infectious and thus they might affect transmission efficiency, while microbes colonising the *Anopheles* genitalia may impact reproductive success, immunity, lifespan and microbiota composition of the offspring. The endosymbiont *Wolbachia* is found in half of the insect species, but had not been detected in any *Anopheles* species until recently. When artificially introduced in an *An. stephensi* strain, *Wolbachia* persists in mosquito ovaries, is vertically transmitted and increases the resistance against *P. falciparum* in laboratory conditions [60]. *Wolbachia* has now been found associated with around 10% of *Anopheles* in a wild population in Burkina Faso [61]. Its load was, however, much lower than that of other insect species, notably *Ae. albopictus* where *Wolbachia* was found to account for 99% of the *16S* rRNA reads in whole mosquitoes [40, 61–63]. It was proposed that some members of the ovary microbiota, notably *Asaia*, interfere with *Wolbachia* colonisation and thus reduce its possible use against malaria [64, 65].

The microbiota of the ovaries and salivary glands has been investigated in *Anopheles* mosquitoes in few metagenomics studies [3, 37, 63, 66]. A recent study of the microbiota composition in midguts, salivary glands and ovaries suggests that individual history rather than the tissue shapes the mosquito microbiota, but still identified some differences in taxon abundance among the three tissues [3].

Functional studies on the role of the microorganisms colonising other tissues beyond the midgut will help to clarify several aspects of *Plasmodium* transmission and mosquito ecology. Moreover, the isolation of bacteria able to colonise the body of the mosquito without affecting its lifespan and to be efficiently vertically transmitted will identify new paratransgenesis candidates able to spread efficiently in the mosquito population. Recently, *Serratia* was identified in both ovaries and salivary glands of *Anopheles* mosquitoes and proposed for malaria control [3, 8].

## Conclusions

In the past 20 years, the pivotal role of the mosquito microbiota in shaping *Plasmodium* infection and transmission has gradually emerged. However, the tripartite interaction between the mosquito, its microbiota and the parasite is a complex relationship that still needs further investigation. In general, the microbiota was found to reduce *Plasmodium* infection and to impact several physiological aspects of the mosquito, notably affecting its lifespan. Surprisingly, these effects induced by the microbiota were consistent almost irrespective of the *Anopheles* and *Plasmodium* species, suggesting that this tripartite interaction is a stable system in which each component plays a role. Although our knowledge on the mosquito microbiota is continuously expanding, several aspects have not been completely elucidated yet and represent the current challenges of this field. In particular, the non-bacterial component of the mosquito microbiota has not been investigated as extensively as the bacterial one, although viruses and eukaryotes might be as relevant as prokaryotes in limiting *Plasmodium* infection. Moreover, it is not clear whether the microbiota of the reproductive track or salivary glands impacts parasite transmission or mosquito fitness. Finally, most of the functional studies conducted on the mosquito microbiota have been carried out on laboratory-reared insects, which are known to possess a different microbial community from that of field mosquitoes. Depending on the desired levels of control on experimental conditions and of relevance of microbiota composition, several experimental set ups may be used to improve the study of the mosquito microbiota.

## Abbreviations

Duox: Dual oxidase
IAFGP: *Ixodes scapularis* antifreeze glycoprotein
Imd: Immune-deficiency
IMPer: Immunomodulatory peroxidase
OTU: Operational taxonomic unit
PGRP: Peptidoglycan recognition protein
ROS: Reactive oxygen species
TEP1: Thioester-containing Protein 1
